# Prevalence of Use of Preventive Services in Poland: Result from a Population-Based Nationwide Study

**DOI:** 10.3390/jcm10102084

**Published:** 2021-05-12

**Authors:** Siddarth Agrawal, Justyna Gołębiowska, Sebastian Makuch, Grzegorz Mazur

**Affiliations:** 1 Department and Clinic of Internal Medicine, Occupational Diseases, Hypertension and Clinical Oncology, Wroclaw Medical University, 50-556 Wroclaw, Poland; ju.golebiowska@gmail.com (J.G.); grzegorz.mazur@umed.wroc.pl (G.M.); 2 Department of Cancer Prevention and Therapy, Wroclaw Medical University, 50-556 Wroclaw, Poland; 3 Department of Pathology, Wroclaw Medical University, 50-368 Wroclaw, Poland; sebastian.mk21@gmail.com

**Keywords:** clinical preventive services, disease prevention, public health

## Abstract

Systematic reviews of scientific evidence have identified clinical services that prevent or ameliorate illness and reduce mortality. This study aimed to assess the prevalence of all recommended evidence-based preventive services in a publicly funded healthcare setting. We conducted a population-based nationwide cross-sectional computer-assisted telephone survey of 1000 Polish adults (response rate 42%). The self-reported use of all recommended clinical preventive services was assessed, including mammography, colonoscopy, blood glucose screening, vaccination, blood pressure screening, and preventive counselling. The results showed that only 6.4% of adults had received all recommended preventive screening, whereas only 4.3% had received appropriate counselling. General practitioner (GP) visits, blood pressure screening, blood glucose screening, and cervical smear were among the most commonly provisioned interventions, while flu vaccination, PSA assessment, and preventive counselling were among the least prevalent services. Despite the low uptake of preventive interventions, over 75% is interested in remote access to preventive services using telemedicine platforms and e-consultations. Our findings suggest that there are significant gaps in the receipt of preventive interventions. Further improvements require not only changes in the incentive system for healthcare providers, but also system-level innovation such as telemedicine solutions to deliver preventive services remotely and engage individuals in the monitoring process.

## 1. Introduction

Scientific evidence based on systematic reviews has identified clinical services that lead to disease prevention and mortality reduction [1,2,3]. Despite the availability of these evidence-based tools and the existing data regarding their economic viability, previous assessments have demonstrated significant gaps in their receipt. For example, less than half of Europeans receive cancer screening services and preventive counselling [4,5]. Most prior studies have considered the provision of a single domain of preventive services (such as vaccination or cardiac care) and failed to evaluate a systemic approach to preventative care. A recent study by Borsky et al. has shown that only 8% of adult Americans receive all high-priority, appropriate clinical preventive services recommended for them [6]. To date, there are no studies to show the level of receipt of all recommended preventive services in a publicly funded healthcare setting. In this population-based nationwide cross-sectional study, we assessed the utilization of all appropriate clinical preventive services by adults in a publicly financed healthcare system in Poland.

## 2. Materials and Methods

### 2.1. Database

A nationwide cross-sectional study was carried out in May–June 2020 on a representative sample of 1000 Polish adults aged 18 years or older using computer-assisted telephone interviews (response rate 42%). 

### 2.2. Study Population

A stratified sampling per the demographic structure of voivodeships (the highest-level administrative division of Poland) was used to obtain a representative sample of the population. Target quotas were set for age and gender strata in each geographical region. The interviewers were adequately trained and prepared for the application of the study survey to ensure quality. A data collection supervisor supervised all interviews, and a study coordinator randomly evaluated the recordings of the conversation. The transcripts were not returned to participants for comment and/or correction. No repeat interviews were carried out. The average duration of the interview was 15 min. 

Participants provided their verbal consent at the beginning of the interview. No compensation was provided for participating in the study. The study was approved by the Bioethics Committee of Wroclaw Medical University.

### 2.3. Variables

The study survey asked questions about the utilization of thirteen preventive services that were identified based on an expert review of national recommendations. Preventive services were classified into two groups: (1) Preventive screening and (2) preventive counselling. All recommended preventive services for specific age and gender groups, as well as the reference period for each service, are listed in Appendix A, Table A1. The study survey is available in Appendix B. The total number of recommended services differed for each person based on age, gender, and medical information. The minimum number of services one should have utilized is 7, while the maximum is 12. On average, each individual should have received 9 services. These services are accessible, free of charge, and covered by public funds. Health promotion programs have been created using the recommendations of the Polish National Health Program. 

### 2.4. Statistical Analysis

We employed a composite measure to evaluate whether an individual received all appropriate preventive services according to a specific age and gender group. Two data coders coded the data. Statistical analysis was performed using the Statistica v.13.3 (StatSoft). The normality of quantitative variables was verified with the Shapiro-Wilk test. Due to the lack of normal distribution, the statistical significance of differences between the two services groups was assessed using the non-parametric Mann-Whitney U test. Qualitative variables are presented in the contingency tables in the form of counts (n) and fractions (%). The independence of two qualitative variables was verified using the Pearson Chiquadrat test. Whenever statistical hypothesis testing was used, a p-value of less than 0.05 was considered statistically significant. 

## 3. Results

**Figure 1:**
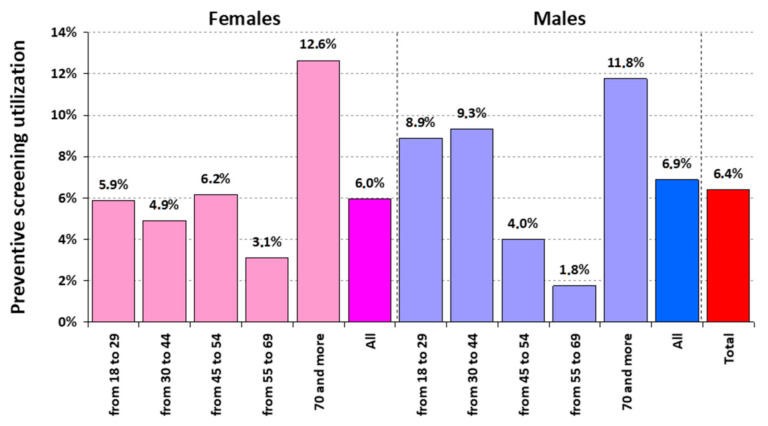
Percentage of adults receiving all recommended preventive screening, by gender and age.

The study included 1000 participants (520 females and 480 males) over 18 years of age (mean age 47 years, SD = 17 years). All clinical preventive services were divided into two groups, preventive screening (cancer screening, vaccination, blood pressure assessment, etc.) and preventive counselling (for obesity, alcohol abuse, tobacco cessation, and depression). Overall, 6.4% (95% CI: 4.88, 7.92) of adults had received preventive screening. General practitioner (GP) visits, blood pressure screening, blood glucose screening, and cervical smear were among the most commonly provisioned interventions, at more than 60%. In contrast, flu vaccination and PSA assessment were the least frequently received screening tools. Women were more likely to receive most of the screening interventions. These differences reached statistical significance for lipid screening, colonoscopy, and blood glucose screening (Table 1). However, men were more likely to receive flu vaccination than women (15% vs. 10.8%; *p* < 0.047), while among 215 people being above the age of 65, 23 out of 37 participants that received influenza vaccine were women. The percentage of adults receiving all recommended screening interventions by gender and age is presented in Figure 1. In both genders, older people (aged 70 or more) were almost twice more likely to receive preventive screening than average adults.

Preventive counselling was significantly less utilized when compared to preventive screening. In total, only 4.3% of all adults received appropriate counselling based on their medical information. Strikingly, only 20.3% of adults with BMI greater than 25 kg/m^2^ received obesity counselling, and less than 9% of alcohol abusers were counselled for alcoholism. Interestingly, men were more likely to receive preventive counselling than women. The difference between these two groups reached statistical significance for alcohol and nicotine abuse counselling (Table 1).

**Figure 2:**
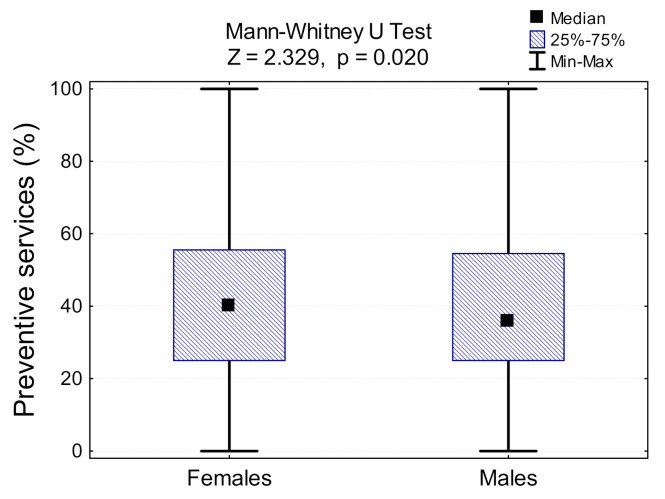
Percentage of preventive services received by adults in groups differing between genders.

**Figure A1:**
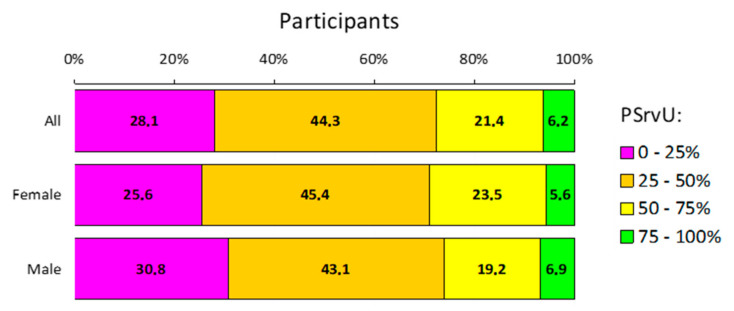
Percentage of adults receiving all recommended, appropriate clinical preventive services, by gender and quartile of service receipt. PSrvU—preventive service utilization.

**Figure A2:**
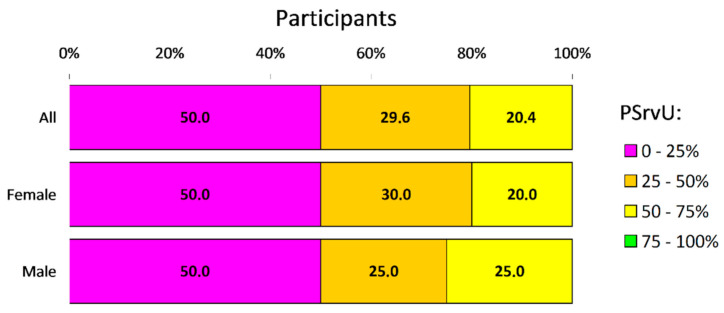
Percentage of adults receiving all recommended, appropriate clinical preventive services, excluding flu vaccination for the entire adult population and PSA-testing, by sex and quartile of service receipt.

Overall, only 1.5% (95% CI: 0.75, 2.25) of adults received all appropriate, recommended clinical services (both screening and counselling). Table 2 shows the percentage and 95% confidence interval of the adult population receiving all preventive services, by gender. Given the low rate of the respondents who had received all of the preventive interventions, we examined the percentages of screening and counselling tools that adults had received. Females were more likely than males to receive preventive services (40.0% vs. 36.0%; *p* = 0.02; Figure 2). When we excluded flu vaccination and PSA-testing from the analysis, the percentage of services that females had received was higher than men (80.0% vs. 74.3%; *p* = 0.198; Appendix A, Table A2, Figure A1 and Figure A2).

We have assessed the respondents’ expectations for the delivery of preventive services. Nearly three-quarters of the respondents expect the public health system to provide access to preventive services. Interestingly, females show significantly higher rates of expectancy than males (76.5% vs. 68.3%, *p* = 0.05). Moreover, over 75% of men and women are interested in remote access to preventive services using telemedicine platforms and e-consultations (Table 3).

## 4. Discussion

Our study explored the utilization of an evidence-based package of all recommended preventive services in a publicly financed healthcare system. Our findings suggest that there are significant gaps in the receipt of appropriate preventive interventions. The results are consistent with findings from previous studies that evaluated the uptake of individual preventive services [4,5,7]. We have found that the receipt of preventive screening was highest in older adults aged 70 or more. The finding may be linked with the fact that the elderly are more likely to receive clinical care and medical advice, both in hospital as well as a primary care setting, as well as the fact that they have more free time to attend screening, compared to the younger population [8,9]. The uptake of cancer screening services, such as colonoscopy and PSA testing, was surprisingly low, at 15.2% and 26.2%, respectively. We have found significant gaps even among the highly-utilized services such as GP visits, blood pressure screening, cervical smear, and blood glucose screening, where nearly a third of the population had not received preventive care.

Preventive counselling (referring to obesity, alcohol abuse, tobacco cessation, and depression), which ranks among the top cost-saving interventions [10], is delivered to less than a quarter of the population. This result highlights a wide gap in the use of these impactful interventions. The projections show that a higher uptake of preventive counselling for tobacco cessation, alcohol misuse, depression, and obesity would add over 1,000,000 QALYs and save billions of dollars [10,11]. 

The differences in the receipt of preventive services among men and women were significant. Females were significantly more likely to receive laboratory tests (blood glucose, lipid profile) and colon cancer screening. These results are consistent with previous findings, which indicate that women have a higher medical care service utilization than men [6,12]. 

Overall, almost 75% of all adults expect the public health system to provide them with all recommended preventive services. The metric shows a high interest among individuals in preventive care. Moreover, in our study, the respondents were willing to employ telemedicine solutions to access preventive care remotely. These results may indicate that a higher utilization rate could be achieved by improving health communication and using new channels of service delivery. 

The study is subject to limitations. First of all, we obtained a relatively low response rate (42%). Low response rates are commonly considered as a limitation in population-based nationwide studies and may constitute a source of selection bias. Moreover, receipt of preventive services was self-reported and may be subject to recall bias. Self-report data tend to overreport utilization rates [13]. However, in our study, rates of service use were consistent with estimates from European data [7]. Secondly, while the results are nationally representative, the sample size did not facilitate more analyses of disparities in receipt of preventive services. Thirdly, to select a representative sample of the Polish adult population, a stratified sampling per the demographic structure of voivodeships was used. Additionally, we failed to report on the overuse of preventive services, as well as on the proportion of people who appropriately chose not to get services that may have been available to them. However, we set target quotas for age and gender strata in each geographical region. Therefore, the inherent limitations of quota-sampling are present.

Projections of future morbidity and burden of disease indicate that chronic illness will continue to be the most significant contributor to mortality and disability in Europe and high-income countries [14]. It is estimated that almost nine out of ten deaths in the European Union are due to chronic diseases, including cancer, cardiovascular disease, diabetes, and mental illness [15]. The financial load linked with the management of chronic diseases is enormously high, and given that the burden of chronic diseases is continuously increasing, chronic illness will continue to put heavy pressure on national economies. Today, more than 50 million people in Europe have multiple chronic conditions, which incur even higher costs of care and treatment [16]. In the times of COVID-19 pandemic, it is intriguing how much is being spent on preventive care to reduce the prevalence of the diseases [17]. On average in the EU, based on both public and private healthcare, the expenditure on preventive care was estimated at 2.8% of total health expenditure in 2018 [18]. The highest shares were recorded in Italy (4.4%), while the lowest percentage of preventive care expenditure was recorded in Slovakia (0.8% of total health expenditure) [7,18]. Poland was ranked 15th out of 27 countries of EU, with a share of preventive care expenditure accounting for 2.3% [18]. However, taking into account the population size of each EU country, the preventive care expenditure was highest in Sweden (USD 165 per inhabitant) and lowest in Romania and Slovakia (both USD 8 per inhabitant), while Poland was ranked 23rd (USD 19 per inhabitant) [7,18]. This is an indication that an increase of clinical preventive services delivery in Poland is in dire need.

Clinical preventive strategies are available for many chronic diseases and their value, both health impact and cost-effectiveness remain consistent [10]. Projections show that investment in a high-priority evidence-based package of preventive interventions for the population would produce over 2 million additional years of life each year they are provisioned [11]. For example, preventive counselling for tobacco use, alcohol abuse, and depression, which proved to be significantly underutilized in our study, are an expected cost-saving service [10]. Increasing the receipt of evidence-based preventive services results in a reduction of complications of the illness, long-term healthcare costs, and premature deaths.

Despite the mounting evidence, the uptake of preventive services is surprisingly low. The primary reason includes a low level of public awareness about strongly recommended preventive services, gaps in provider capacity, including long waiting times, and higher focus on diagnosis and provision of treatments rather than preventive interventions among healthcare providers [5]. A recent study has shown that both medical personnel and administrative stakeholders are aware of the health and economic benefits of disease prevention [19]. It is assumed that the low uptake of the preventive services is due to an implementation gap, which is caused by a lack of financial incentives for medical providers to prevent chronic illness. To date, the majority of providers, in particular hospitals and medical professionals, are paid to manage rather than to prevent disease.

In conclusion, despite the current limitations, comprehensive preventive care is attainable. Our data indicate that almost a third of adults reported utilizing more than half of the recommended preventive interventions, and only 0.7% had not received preventive care at all (Appendix A, Table A2, Figure A1 and Figure A2). Services that are most commonly not being delivered, such as preventive counselling, need to be emphasized to achieve greater coverage of the population. Further improvements require not only changes in the incentive system for healthcare providers, but also system-level innovation such as telemedicine solutions to deliver preventive services remotely and engage individuals in the monitoring process. A systemic and rational approach to ensuring that all individuals receive evidence-based preventive services is urgently needed. The effective preventive strategy will attain the multiple objectives of improving the quality of life, extending the human lifespan, and making the best use of scarce resources.

## Figures and Tables

**Table 1:** Percentages of adults receiving recommended clinical preventive services, by gender.

Preventive Services	All	Females	Males
Screening			
GP visit	73.3	75.6	70.8
Blood pressure	67.8	67.7	67.9
Flu vaccination	12.8	10.8	15 *
Lipid profile	59.1	64.9	52.9 ***
Colonoscopy	19.1	22.7	15.2 **
Blood glucose	65.8	71.3	59.9
Cervical smear	67	67	-
Mammography	51	51	-
PSA	26.2	-	26.2
Counselling			
Obesity	20.3	19	21.7
Alcohol consumption	8.9	6.2	11.9 **
Tobacco use	17.4	13.7	21.5 **
Depression	19.8	19.8	19.8

Significance refers to the difference between females and males. PSA is prostate-specific antigen. - Not applicable. * *p* < 0.05, ** *p* < 0.001, *** *p* < 0.0001.

**Table 2:** Percentage and 95% confidence interval of respondents receiving all preventive services, by gender.

	All	Females	Males
Preventive screening utilization = 100%	6.40 [4.88, 7.92]	5.96 [3.92, 8.00]	6.88 [4.60, 9.15]
Preventive counselling utilization = 100%	4.30 [3.04, 5.56]	3.08 [1.59, 4.57]	5.62 [3.56, 7.69]
Preventive service utilization = 100%	1.50 [0.75, 2.25]	0.58 [0.00, 1.23]	2.50 [1.10, 3.90]

**Table 3:** Respondents expectations for the delivery of preventive services, by gender.

	Female N = 520		Male N = 480		All N = 1000		*p*-Value
	N	%	N	%	N	%	
Do you expect the public health system to give you access to preventive services and provide all necessary information?							0.005
Yes	398	76.5%	328	68.3%	726	72.6%	
No	122	23.5%	152	31.7%	274	27.4%	
Would you be interested in telehealth solutions to access preventive services remotely (e.g., via telemedicine platforms, e-consultations, etc.)							0.426
Yes	398	72.6%	356	74.2%	754	75.4%	
No	122	23.5%	124	25.8%	246	24.6%	

## Data Availability

The authors confirm that the data supporting the findings of this study are available within the article.

## Appendix A

**Table A1:** All recommended preventive services.

Preventive Services	Reference Period	Target Group	Strength of Recommendation
Blood pressure	annual	all	High
Blood sugar	every three years	adults aged 45 to 69	High
Lipid profile	every five years	females aged 45 to 69 men aged 35 to 69	High
Flu vaccination	annual	all	Low
Colonoscopy	every ten years	adults aged 55 to 64	High
GP visit	annual	all	High
Obesity counselling	annual	adults with a BMI of 25 kg/m^2^ or more	Low
Alcohol consumption counselling	annual	for alcohol abusers	Low
Tobacco use counseling	annual	for smokers	Low
Depression counselling	annual	all	Low
PSA measurement	annual	males aged 50 to 69	Low
Mammography	every two years	females aged 50 to 69	High
Cervical smear	every three years	females aged 25 to 39	High

**Table A2:** Percentage of respondents in groups differing in the utilizaition of preventive services (PSrvU), preventive screening (PScrU), and preventive counselling (PCnsU).

Percentage of Utilization	All Percentage of Participants (*n*)			Quartiles	All Percentage of Participants (*n*)		
	PScrU	PCnsU	PSrvU		PScrU	PCnsU	PSrvU
0%	0.7 (7)	61.8 (618)	0.7 (7)	0–25	15.0 (150)	72.4 (724)	28.1 (281)
>0%, ≤25%	14.3 (143)	10.6 (106)	27.4 (274)				
>25%, ≤50%	25.4 (254)	15.2 (152)	44.3 (443)	>25–50	25.4 (254)	15.2 (152)	44.3 (443)
>50%, ≤75%	43.3 (433)	7.4 (74)	21.4 (214)	>50–75	43.3 (433)	7.4 (74)	21.4 (214)
>75%, <100%	9.9% (99)	0.7 (7)	4.7 (47)	>75–100	16.3 (163)	5.0 (50)	6.2 (62)
100%	6.4% (64)	4.3 (43)	1.5 (15)				
**Percentage of Utilization**	**Females Percentage of participants (*n*)**			**Quartiles**	**Females Percentage of participants (*n*)**		
	**PScrU**	**PCnsU**	**PSrvU**		**PScrU**	**PCnsU**	**PSrvU**
0%	0.2 (1)	64.8 (337)	0.2 (1)	0–25	12.9 (67)	74.2 (386)	25.6 (133)
>0%, ≤25%	12.7 (66)	9.4 (49)	25.4 (132)				
>25%, ≤50%	24.4 (127)	16.5 (86)	45.4 (236)	>25–50	24.4 (127)	16.5 (86)	45.4 (236)
>50%, ≤75%	46.2 (240)	5.2 (27)	23.5 (122)	>50–75	46.2 (240)	5.2 (27)	23.5 (122)
>75%, <100%	10.6 (55)	1.0 (5)	5.0 (26)	>75–100	16.5 (86)	4.0 (21)	5.6 (29)
100%	6.0 (31)	3.1 (16)	0.6 (3)				
**Percentage of Utilization**	**Males** **Percentage of Participants (*n*)**			**Quartiles**	**Males** **Percentage of Participants (*n*)**		
	**PScrU**	**PCnsU**	**PSrvU**		**PScrU**	**PCnsU**	**PSrvU**
0%	1.3 (6)	58.5 (281)	1.3 (6)	0–25	17.3 (67)	70.4 (338)	30.8 (148)
>0%, ≤25%	16.0 (77)	11.9 (57)	29.6 (142)				
>25%, ≤50%	26.5 (127)	13.8 (66)	43.1 (207)	>25–50	26.5 (127)	13.8 (66)	43.1 (207)
>50%, ≤75%	40.2 (193)	9.8 (47)	19.2 (92)	>50–75	40.2 (193)	9.8 (47)	19.2 (92)
>75%, <100%	9.2 (44)	0.4 (2)	4.4 (21)	>75–100	16.0 (77)	6.0 (29)	6.9 (33)
100%	6.9 (33)	5.6 (27)	2.5 (12)				

## Appendix B. Study Survey (Translated From Polish)

1. What is your weight (in kilograms)?
2. What is your height (in centimeters)?
3. When was the last time you visited a doctor for a health assessment, follow-up care for an ongoing problem, or a concern that you have about your health? Do not include emergency visits or hospitalizations.
  - within the past 12 months
  - within the past 1 to 2 years
  - within the past 2 to 5 years
  - more than five years ago
  - never
4. Have you been vaccinated against the flu within the previous 12 months?
  - Yes
  - No
5. During the past 12 months, has doctor or other healthcare professional given you advice about how to manage your weight, discussed weight loss goals with you, or referred you to a weight loss program to help with your diet and exercise?
  - Yes
  - No
6. Within the past 12 months, has doctor or other healthcare professional asked you how much and how often you drink alcohol? You may have answered in person, on paper, or on a computer.
  - Yes
  - No
7. Within the past 12 months, have you had 4 or more standard portions of alcohol? (A standard portion refers to 250 mL glass of 5% beer, 100 mL glass of 12% wine or 30mL glass of 40% vodka)
  - Yes
  - No
8. Within the past 12 months, has doctor or other healthcare professional advised you to cut back or stop drinking alcohol?
  - Yes
  - No
9. Within the past 12 months, on average, would you say you smoked cigarettes or used tobacco every day, some days, or not at all?
  - Every day
  - Occasionally
  - Not at all
10. Within the past 12 months, has doctor or other healthcare professional advised you to quit smoking or quit using tobacco?
  - Yes
  - No
11. Within the past 12 months, has doctor or other healthcare professional asked you about your mood, such as whether you are anxious or depressed? You may have answered in person, on paper, or on a computer.
  - Yes
  - No
12. Within the past 12 months, have you had your blood pressure checked by a doctor, nurse, or other health care professional?
  - Yes
  - No
13. Do you expect public health system to give you access to preventive services and provide all necessary information?
  - Yes
  - No
14. Would you be interested in telehealth solutions to access preventive services remotely (e.g., via telemedicine platforms, e-consultations, etc.)
  - Yes
  - No

**Sex specific**

Women:

15. Within the past five years, have you had your blood lipid profile tested?
  - Yes
  - No
16. Have you had a hysterectomy or have you ever had cervical cancer?
  - Yes
  - No
17. Within the last three years, have you had a cervical smear test?
  - Yes
  - No
18. Have you had both breasts removed or have you ever had breast cancer?
  - Yes
  - No
19. Within the last two years, have you had a mammography? A mammography is an x-ray taken only of the breast by a machine that presses against the breast?
  - Yes
  - No
20. Have you had colon cancer or your entire colon removed (colectomy)?
  - Yes
  - No
21. Within the past ten years, have you had a colonoscopy?
  - Yes
  - No
22. Do you have diabetes?
  - Yes
  - No
23. Within the last three years, have you had a blood glucose test?
  - Yes
  - No

Men:

24. Within the past five years, have you had your blood lipid profile tested?
  - Yes
  - No
25. Have you had colon cancer or your entire colon removed (colectomy)?
  - Yes
  - No
26. Within the past ten years, have you had a colonoscopy?
  - Yes
  - No
27. Do you have diabetes?
  - Yes
  - No
28. Within the last three years, have you had a blood glucose test?
  - Yes
  - No
29. Have you ever had prostate cancer?
  - Yes
  - No
30. During the past 12 months, have you had a PSA test?
  - Yes
  - No
